# Static Behavior of a 3D-Printed Short Carbon Fiber Polyamide: Influence of the Meso-Structure and Water Content

**DOI:** 10.3390/ma17091983

**Published:** 2024-04-24

**Authors:** Andrea Canegrati, Luca Michele Martulli, Milutin Kostovic, Gennaro Rollo, Andrea Sorrentino, Michele Carboni, Andrea Bernasconi

**Affiliations:** 1 Department of Mechanical Engineering, Politecnico di Milano, Via La Masa 1, 20156 Milano, Italy; andrea.canegrati@polimi.it (A.C.); michele.carboni@polimi.it (M.C.); andrea.bernasconi@polimi.it (A.B.); 2 Polymer, Composites and Biomaterials Institute, National Research Council (CNR), Via Previati 1/E, 23900 Lecco, Italy; milutin.kostovic@ipcb.cnr.it (M.K.); gennaro.rollo@ipcb.cnr.it (G.R.); andrea.sorrentino@cnr.it (A.S.)

**Keywords:** fused-filament fabrication, 3D printing, short fiber reinforced polyamide, material characterization, failure analysis

## Abstract

The knowledge of the mechanical behavior of a 3D-printed material is fundamental for the 3D printing outbreaking technology to be considered for a range of applications. In this framework, the significance, reliability, and accuracy of the information obtained by testing material coupons assumes a pivotal role. The present work focuses on an evaluation of the static mechanical properties and failure modes of a 3D-printed short carbon fiber-reinforced polyamide in relation to the specimen’s unique meso-structural morphology and water content. Within the manufacturing limitations of a commercially available printer, specimens of dedicated combinations of geometry and printing patterns were specifically conceived and tested. The specimens’ meso-structure morphologies were investigated by micro-computed tomography. The material failure mechanisms were inferred from an analysis of the specimens’ fracture surfaces and failure morphologies. The outcomes of the present analysis indicate that each test specimen retained proper mechanical properties, thereby suggesting that they should be accurately designed to deliver representative information of the underlying material beads or of their deposition layout. Suggestions on the adoption of preferred test specimens for evaluating specific material properties were proposed.

## 1. Introduction

Fused-filament fabrication (FFF) is a 3D printing technology that has recently drawn great interest due to the prospect of a mold-less, one-step production of highly customizable functional parts that are tailored to customer needs. In FFF, the printer extrudes beads of semi-molten thermoplastic polymer through a nozzle. The nozzle travels along prescribed planar trajectories and deposits them on a building platform in a layer-wise fashion. Short fiber-filled thermoplastic polymers can be processed without radical changes in the FFF printer layout [1]. This could make fused-filament-fabricated (FFF-ed) Short Fiber-Reinforced Polymers (SFRPs) infill material candidates of 3D-printed structural parts [2,3]. However, their mechanical properties still require a deep investigation to fully understand their potential fields of application.

The macroscopic mechanical properties of FFF-ed SFRPs are determined by the combination of their micro-structural architecture and their inherent unique meso-structure, which is induced in each singular 3D-printed material coupon by the deposition strategy of the printer. The mechanical properties of the extruded material bead may differ from those of the feedstock material filament. The extrusion process favors a preferential alignment of the short fibers along a bead’s longitudinal axis. Concurrently, it damages the fibers, reducing their aspect ratio and consequently their reinforcement effectiveness in the composite [4]. Additionally, the meso-structure of a material coupon generally features voids and printing defects, which are expected to act as possible stress concentrators and possibly cause premature failures [4]. 

Unidirectional (UD) specimens, displaying material beads entirely deposited at 0° or 90°, have been extensively used in the literature to characterize the transversal isotropic mechanical properties of FFF-ed SFRPs [1,4,5]. Nevertheless, the highly discontinuous morphology of a specimen’s cross section makes the estimation of the underlying material bead’s properties challenging [4]. Additionally, the transverse mechanical properties appear to be mainly governed by the quality of the adhesion between adjacent intra or inter-layer beads [4]. Therefore, by testing the UD 90° specimens, the bond properties rather than the transversal material properties are likely characterized.

Moreover, several FFF printers on the market provide limited control over the deposition strategy of each individual layer and impose part contouring [6], which is where at least one contour bead should be deposited to shape the outline of the layers and then the inner space is filled with beads of prescribed patterns. Contour beads, in general, display different orientations than that of infill ones. For the present investigation, a Markforged^®^ printer and its own marketed Short Carbon Fiber-Reinforced polyamide 6 (SCFR-PA6), best known under the tradename of Onyx [7], was considered as a representative instance of FFF-ed material characterization within the framework of manufacturing restrictions imposed by this class of printers. With their default settings, Markforged^®^ printers do not allow UD specimens to be printed flat on the building platform [8]. If a solid (rectilinear) 100% dense infill is desired, Eiger, the proprietary slicing software by Markforged^®^, stacks slices with an alternatively perpendicular sequence of a raster orientation [8]. This setting cannot be edited by the user. This further hinders the characterization of the mechanical properties of the underlying material [9], thus suggesting that a multi-scale analysis is required to link the macroscopic properties of a material coupon to those of the material itself.

Several studies have dealt with analogous combinations of materials and printers, wherein different specimen geometries and printing strategies for material characterization have been investigated. Kubota et al. [8] estimated the SCFR-PA6’s transversally isotropic tensile properties by testing prismatic FFF-ed 0° and 90° UD specimens. However, the authors applied unconventional deposition strategies, based on a deposition of contour beads only, and a second manufacturing step to obtain such specimens. Fisher et al. [10] characterized the longitudinal tensile properties of SCFR-PA6 by testing FFF-ed ASTM D638 [11] standard-complaint dog bone specimens in two configurations: flat specimens, where their gauge length consisted of contour beads only (all parallel to the loading direction), and on-edge 0° UD specimens, which consisted of all contour beads. Nevertheless, both specimens experienced defect-induced premature failure. SCFR-PA6 prism-shaped compression specimens were FFF-ed and tested in accordance with the ASTM D695 [12] standard by Fisher et al. [10]. To estimate the transversal isotropic material compressive properties, such specimens were printed by contour beads only in the two following orientations: all the beads of a layer being either parallel or perpendicular with the loading direction. An increase of 130% in the compressive modulus of the parallel printing configuration over the perpendicular one was found. However, the specimen with beads parallel to the load experienced buckling.

The estimated transversally isotropic material properties might be either useful in simulations where the behavior of material beads is explicitly considered, or for the micromechanical material model calibration. However, workpieces will be likely FFF-ed by the default settings of the printer, thus displaying the sequential mutually perpendicular raster orientation, in principle, at any possible angle to the load. Therefore, the mechanical properties of specimens displaying such meso-structural layer arrangement might be representative of those of a workpiece with a similar layout. One might think to use them as homogenized (macroscopic) mechanical properties in numerical simulations. 

Several authors have investigated the tensile properties of SCFR-PA6 specimens FFF-ed under the Markforged^®^ printers default settings. The focus is here addressed to 100% dense solid infills. Kubota et al. [8] tested contour-free specimens with a ±45° raster orientation by cropping them out from larger plates. A tensile modulus of 0.74 GPa, a strength of 32 MPa, and a strain at the break of 58% of such specimens were obtained. Pascual-Gonzalez et al. [13] also tensile tested SCFR-PA6 dog bone specimens with a ±45° raster orientation; however, no information was provided about the contouring of the specimens. They reported a Young’s modulus of 1.2 GPa and a tensile strength of about 53 MPa at 38% of strain. Fisher et al. [10] tensile tested specimens printed with a default infill deposition strategy (±45° raster orientations) and two contour beads. About 0.75 GPa of tensile modulus, 28 MPa of strength, and 75% of failure strain were reported. 

Majko et al. [14] investigated the effect of 0°/90° and ±45° raster orientations (among others) and a number of contour beads on the tensile properties of SCFR-PA6 dog bone specimens. For specimens with the same contour beads, the tensile strength and the failure strain of the ±45° raster orientation were higher than that of the 0°/90° one. An increased number of contour beads led to higher strength and lower strain at failure. A strong dependency of the failure mode and the orientation of the fracture surface on the raster orientation were also observed. Nikiema et al. [15] evaluated the tensile properties of ASTM D3039 [16]-compliant SCFR-PA6 specimens printed with 0°/90° and ±45° raster orientations (among others) and four contour beads. Considerably larger failure strain and slightly higher tensile modulus and strength were found for the ±45° specimens. The authors additionally investigated the effect of material exposition to a generical humid environment on its tensile properties. They observed a tensile modulus reduction of 66% for a 2% water that was absorbed on a mass basis. Polyamide, indeed, displays a strong tendency to absorb water from the environment. The absorbed water acts as a plasticizer: water molecules increase polymeric chains mobility [17], thereby implying a significant drop of the polymer mechanical properties [18]. 

Besides the expected heterogeneity of the results, which is promoted by the current absence of standards concerning the static characterization of FFF-ed materials [19,20,21], there is still not a clear understanding on which unambiguous information about the connection between a material and meso-structural bead arrangement a particular specimen configuration conveys.

An extensive study on the characterization of the static mechanical properties and of the predominant failure modes of FFF-ed SCFR-PA6 according to the meso-structural morphological features of the specimens adopted and water content is still missing. The present work addresses this literature gap by emphasizing an underrated aspect: FFF-ed test specimens retain a hierarchical structure, thus implying that the structures at multiple scales collectively influence their macroscopic mechanical properties. Custom-conceived specimens were designed and tested to support the identification of specimens able to deliver specific information to designers, such as the mechanical properties of the underlying material or of a specific bead’s deposition layout (meso-structure). A critical discussion of the evaluated properties was supported by a micro-computed tomography (µ-CT) investigation of the specimens’ meso-structure morphologies and by scanning electron microscopy (SEM), which aided failure analysis. The present study laid the foundation for investigations of the specimen’s meso-structural-dependent fatigue behavior of the FFF-ed SCFR-PA6 material that was carried out in [22].

## 2. Materials and Methods

### 2.1. Specimen Fabrication

All the specimens considered in this work were manufactured on a Markforged Onyx Pro commercial 3D printer and made of Onyx [7]: a polyamide 6 filled with 14–16% by weight chopped carbon fiber [13,23,24]. The length and the diameter of the carbon fibers were approximately 130–140 µm [13,23,25] and 7–8 µm [13,25], respectively. Significantly high-fiber alignment along the deposition direction of the extruded material beads was observed in [24,25]. Therefore, the fiber orientation may be approximated to that of the printing pattern. The Onyx material was extruded at a temperature of 245 °C and deposited onto a non-heated printing bed. The nozzle diameter was 0.4 mm, which was approximately as much as the width of the deposited beads. Thus, the layer height was set to 0.1 mm instead. The default printing speed and material extrusion rate, which are not disclosed by Markforged^®^, as pointed out in [8], were used. 

The current lack of a specific standard that addresses the static characterization of additively manufactured material properties makes the selection of preferred geometries and internal bead layouts for test specimens challenging. A straightforward application of the available standards, ones that are developed for conventionally manufactured polymeric composites [11,12,16,26] to FFF-ed ones, might lead to controversial results [19,21]. Therefore, the transfer of prescriptions reported in the available standards [11,12,16,26] to FFF-ed test specimens for static properties evaluation will be discussed in the forthcoming sections for the purposes of the present investigation.

#### 2.1.1. Tensile Specimens

Test methods for the tensile property evaluation of the polymeric composites are thoroughly described in the ISO 527-2 [26], ASTM D638 [11], and ASTM D3039 [16] standards. The ISO 527-2 [26] and the ASTM D638 [11] standards prescribe the use of dog-bone-shaped specimens, whose widths taper from the grip region toward the gauge length along large fillet radii. However, in that region, the FFF-ed Onyx dog-bone-shaped specimens used in [10,14] featured defects stemming from the sharp changes in the bead deposition paths. They indeed experienced the premature failure ascribed to the defect-induced onset and development of the crack. Consequently, tensile specimens were designed as rectangular prisms according to the guidelines of the ASTM D3039 [16] standard.

Two sets of specimens with 100% dense rectilinear infill layers, displaying alternating raster orientations at ±45° and 0°/90° with respect to the specimen’s longitudinal axis, were printed to assess the influence of the meso-structure on the specimen’s tensile properties.

Moreover, Eiger (2020 release) imposes the part enveloping by a contour shell. The minimum allowable number of two concentric contour beads were selected to this end. This results in lateral contours occurring in the specimen cross sections with a favorable 0° orientation that runs parallel to the specimens’ longitudinal axes.

The specimen width *w* was selected as a manufacturing variable to further investigate the size effect on the tensile properties of a specimen with given meso-structures. Thus, the specimens were 175 mm long, *w* wide, and 3 mm thick. Two values of *w* were considered: 15 mm and 30 mm. In the rest of this work, the specimens will be referred to as W (for “Wide”) or N (for “Narrow”) for specimens having a 30 mm or 15 mm width, respectively, and this will be followed by the numbers “090” or “45” for specimens having raster orientations of 0°/90° or ±45°, respectively. For example, “W45” will indicate a specimen having a width of 30 mm and a raster orientation of ±45°. Identical FFF-ed W090 and W45 specimens, which are considered in the present study, were also used by the authors in [22] to investigate the fatigue behavior of the same SCFR-PA6.

**Figure 1:**
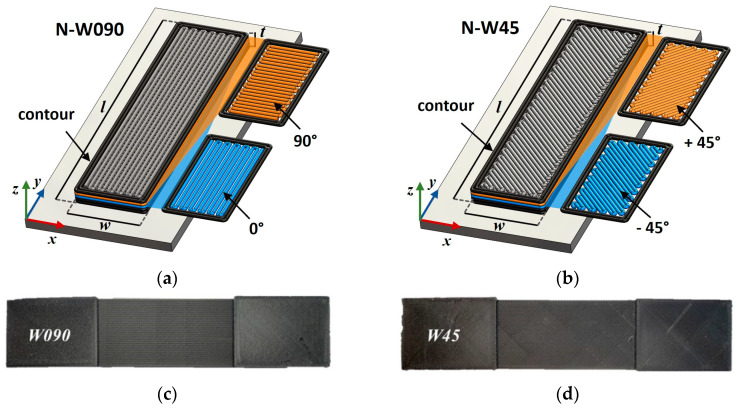
Schematic layout of the tensile specimen with (**a**) 0°/90° orientations, (**b**) ±45° raster orientation, and pictures of (**c**) W090 and (**d**) W45 representative specimens. (Pictures partially taken from [22]).

The layout of the tensile specimens on the building platform, along with their dimensions and a sketch of the mutually perpendicular raster of sequential layers, are reported in Figure 1. The pictures of representative W090 and W45 specimens are reported in Figure 1c and Figure 1d, respectively.

Two batches of tensile specimens were printed. The first batch, consisting of six samples for each specimen configuration, was used to assess the influence of the larger width (size effect) on the specimens’ tensile properties. After printing, these specimens were stored together before testing. While the specimens’ water content was not measured, they always experienced the same environmental conditions. A second batch of specimens, consisting of six W090 and six W45 samples, was used to evaluate the influence of the water content on the specimen’s tensile properties. These specimens were dried in an oven at 70 °C in accordance with the drying procedure used for the water absorption test, which is reported in Section 2.2. After drying, three W090 and three W45 specimens were sealed in waterproof bags until testing. The rest of the specimens were conditioned to their saturation level at 23 °C and a 50% relative humidity before testing.

Onyx end tabs were separately printed with dimensions of 50 mm × *w* × 2 mm. A two-component epoxy adhesive by Pattex^®^ was used to bond the tabs at the ends of the specimens. 

#### 2.1.2. Compression Specimens

Distinct specimen geometries were devised to evaluate the transverse isotropic compressive properties of the FFF-ed SCFR-PA6. Adequate specimens for longitudinal compressive material property characterization feature the stacking of identical layers with unidirectional beads aligned with the load. However, the Eiger software prevents users from printing such unidirectional flat specimens [8]. To partially overcome this limitation, the specimens were printed with a specific deposition strategy, i.e., only concentric contour beads were used [10,27]. This limited the maximum width of the specimens to 12 mm because only a maximum of 15 contour beads could be selected in the slicer software. 

**Figure 2:**
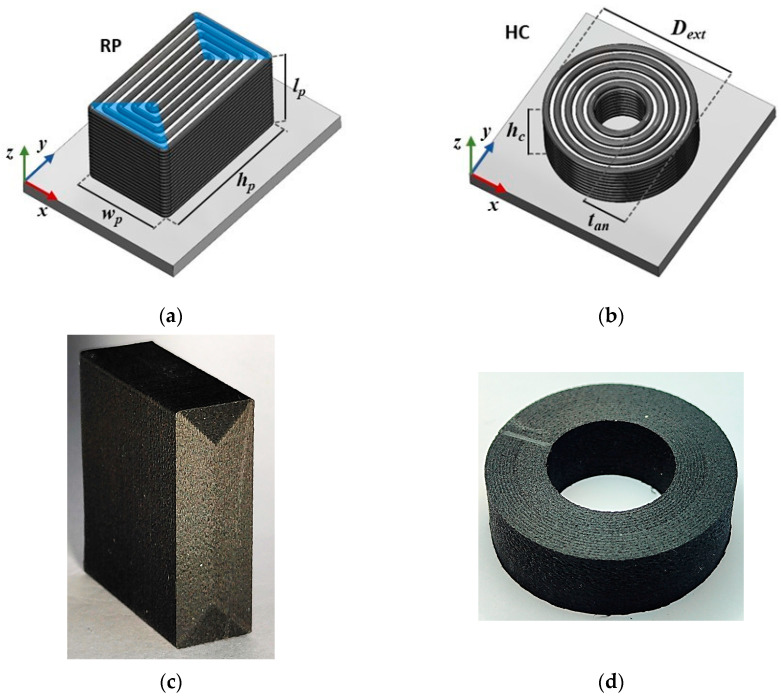
A geometrical sketch of the printing pattern for the (**a**) RP and (**b**) HC specimens. A picture of the actual (**c**) RP and (**d**) HC specimens.

The preferred test specimens suggested in the ASTM D695 [12] standard experienced buckling [10]. Therefore, a slightly different specimen than that recommended in [12] was designed to reduce the chance of buckling to occur. The specimen’s geometry was a rectangular prism (RP), whose dimensions were as follows: a length $l_{p}$ of 30 mm, a height $h_{p}$ of 35 mm, and a width $w_{p}$ of 12 mm. Figure 2a shows the RP specimen’s geometry along with the schematic printing pattern of each layer. The building direction of the specimen, i.e., the *z*-axis of the printer reference frame (Figure 2a), was parallel to the specimen’s length. The testing direction, instead, was parallel to the specimen’s height (the *y* axis of Figure 2a). In Figure 2c, a picture of the RP specimens is shown. The RP specimen displayed a unidirectional bead deposition in the central part of its gauge length. In contrast, at its extremities, wedge-like regions originated from the turns of the printing path close to the specimen edges [10,27]. Beads deposited within these regions ran perpendicular to those outside. These wedge-like regions culminated in sharp corners toward the center of the specimen’s gauge length.

The height of the RP specimens was carefully selected to prevent the occurrence of buckling instability. The specimen was treated as an equivalent column, whose slenderness ratio λ was computed as follows:

(1) λ=leffρgyr,

where $l_{eff}$ is the specimen’s effective length, while $\rho_{gyr}$ is the minor radius of the gyration of the column. As a conservative estimation, $l_{eff}$, was assumed to be equal to the specimen’s height, while for the specimens with a constant rectangular cross-section, $\rho_{gyr}$, was assumed to be 0.289 times the smallest cross-sectional dimension [12]. Overall, for an effective anti-buckling design, the specimens were designed to achieve a slenderness ratio about three times lower than the critical one, $\lambda_{crt}$. This stemmed from the Euler’s formulation of the buckling critical load [28], which reads as follows:

(2) $$\lambda_{crt}=\sqrt{\frac{2\pi^{2}E_{c}}{\sigma_{y}}},$$

where $E_{c}$ and $\sigma_{y}$ are the compressive modulus and yield stress, respectively. However, their values were unknown prior to testing. Therefore, $E_{c}$ and $\sigma_{y}$ were assumed to be equal to the Onyx’s tensile modulus (2.4 GPa) and yield stress (40 MPa) from the Markforged^®^ material datasheet [7], respectively. The resulting slenderness ratio of the RP specimens was about 10, while the critical computed one was about 36. 

For the characterization of the transversal compressive material properties, appropriate specimens should ensure the material’s free and symmetric radial deformation. A rounded shape seems to fit those requirements for the specimens. In the framework of FFF technology, this translates to stacking identical layers with regular and concentric printing paths with respect to the beads transversally aligned to the load. Hollow cylindrical (HC)-shaped specimens were identified as suitable for obtaining specimens made of concentric contour beads only. A full specimen geometry would instead be manufactured by filling the space enclosed by the contour beads with infill beads of predefined raster orientations (±45°), thus hindering the material’s free radial deformation. 

The external diameter $D_{ext}$ of the HC specimens was 30 mm, and the annulus thickness $t_{an}$ was equal to 7.2 mm. The latter was made of 9 contour beads, which were deposited along circular trajectories from the external diameter inward, and 9 similar contour beads were deposited from the internal diameter outward. The specimen height ${\;h}_{c}$, and, more specifically, the specimen’s aspect ratio, was defined as $h_{c}/D_{ext}$, which could affect the barreling phenomenon and consequently the material property characterization. According to [29], the barreling phenomenon is limited for low specimen aspect ratios; hence, $h_{c}$ was set to 10 mm, and was found to yield to an aspect ratio of 0.3. The HC specimen’s geometry is reported on the printing bed in Figure 2b along with the schematic printing pattern of each layer. A picture of the HC specimen is provided in Figure 2d. The testing direction of the HC specimen coincided with its building direction (the z axis of Figure 2b).

### 2.2. X-ray Micro-Computed Tomography and Scanning Electron Microscopy

A GE Phoenix X-ray micro-computed tomography (μ-CT) system, equipped with a flat Dynamic 41|100 digital detector, was used to perform the μ-CT scans of the specimens to examine the morphological features of their meso-structure. The detector offered a 410 × 410 mm^2^ detection area and a 100 μm pixel side size. The entire gauge lengths of two N090, two N45, one W090, one W45, one RP, and one HC were analyzed before testing. The volume reconstructions were carried out with VGSTUDIO MAX 3.4 [30] software by Volume Graphics. The scanning parameters used for each specimen type are reported in Table 1, along with the number of consecutive volumes acquired to frame their entire gauge length.

The fracture surfaces of two tensile specimens per type were observed on a Zeiss EVO 50XVP Scanning Electron Microscope (SEM). A thin gold layer was sputtered on the observed surfaces prior to scanning to enhance the quality of the images due to the low electrical conductivity of the polyamide 6.

### 2.3. Water Absorption Tests

Water absorption tests were performed on one W090, one HC, and one RP. Prior to exposure to the humid environment, all of the specimens were dried in an air-ventilated oven at 70 °C. The specimens were weighted periodically on a scale with a sensitivity of 0.1 mg. The drying procedure ended when no appreciably mass variation was observed over two consecutive days (48 h). After drying, the specimens were placed in a controlled environment within a climatic chamber that is able to maintain the temperature at 23 ± 1 °C and the relative humidity at 50 ± 5%. The specimens’ mass evolution was monitored by periodically applying the same procedure and tools used during drying. The absorption test lasted 35 days (840 h).

### 2.4. Static Tests

Displacement-controlled quasi-static tensile tests were performed on an electro-mechanical MTS RF/100 testing machine equipped with a 100 kN load cell. Tensile tests were performed in two stages. In the first stage, the specimens were loaded at a strain rate of 0.01 min^−1^ up to 0.2 mm. Once this first load ramp was completed, the specimen was unloaded at the same speed and then loaded until failure at a strain rate of 0.05 min^−1^. The speed of the latter ramp was selected as a compromise between a reasonable testing time by considering the large deformation of the tested material, and the reduction in the visco-elastic effects was due to a too high loading rate. The material Young’s modulus was computed from the first ramp in a strain range between 0.05% and 0.25%.

Displacement-controlled quasi-static compression tests were run on an electro-mechanic MTS Alliance RF150 testing machine equipped with a 150 kN load cell. The test strain rate was 0.01 min^−1^. No lubricant was applied at the interface between the compression plates and the testing specimens. Compression tests on the RP specimens were manually interrupted after the detection of a peak load, and those on the HC specimens when the load exceeded a threshold set to 50 kN.

## 3. Results

### 3.1. Morphology of the Specimens’ Meso-Structures

**Figure 3:**
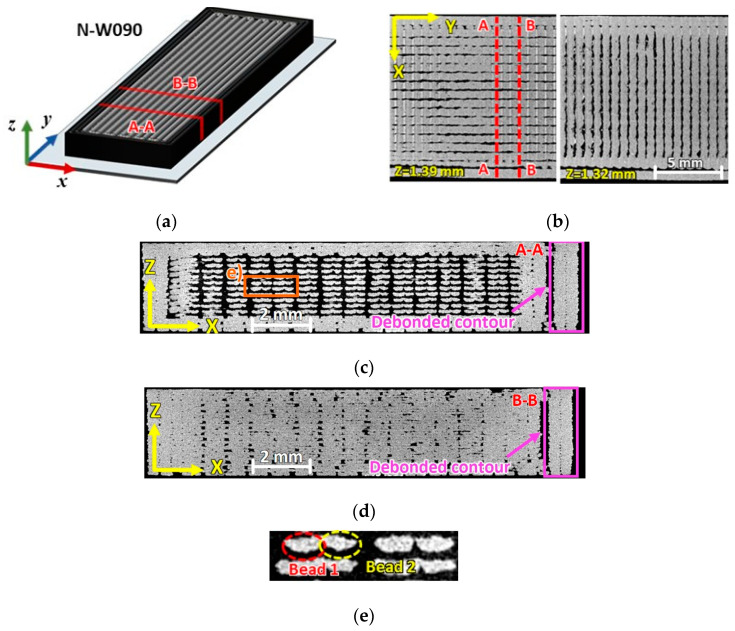
(**a**) Geometry of the 0°/90° specimens highlighting the reference frame, slicing planes (A-A and B-B), and µCT slices of a N090 specimen: (**b**) X-Y layers with 0° and 90° orientations, (**c**) A-A cross-section with high void content, (**d**) B-B cross-section with low void content, and (**e**) close-up on beads.

**Figure 4:**
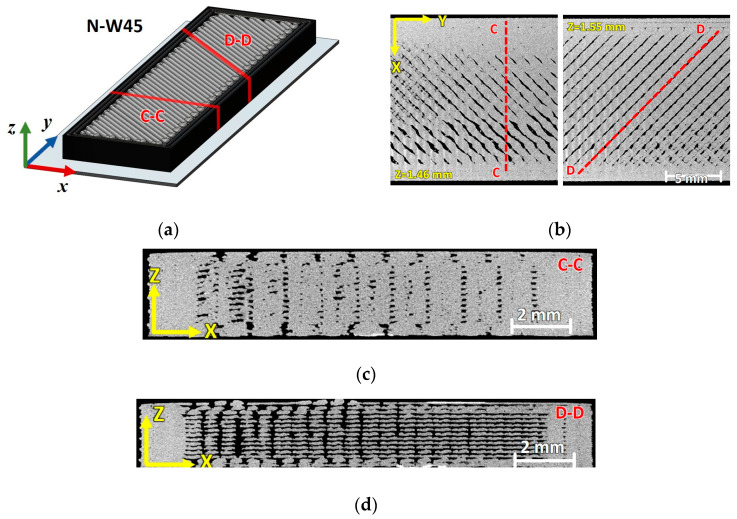
(**a**) Geometry of the ±45° specimens highlighting the reference frame, slicing planes (C-C and D-D), and µCT slices of a N045 specimen: (**b**) X-Y layers with +45° and −45° orientations, (**c**) C-C longitudinal cross-section, (**d**) D-D cross-section at −45°.

The slices extracted from the μ-CT scans of the pristine N090 and N45 representative specimens are shown in Figure 3 and Figure 4, respectively. 

Figure 3a,b and Figure 4a,b highlight the planes over which the μ-CT slices of the specimen cross-sections were extracted. For both raster orientations, no noteworthy difference was observed between the narrow and wide specimens, besides the larger width.

Although a 100% dense infill was selected, a large extent of the intra-bead voids was still present within the 0°/90° specimen, as shown in Figure 3. The voids appeared to be predominantly concentrated in the core region of the specimens, while lateral contours and the roof/floor layers seemed to retain a more compact structure (see Figure 3b–d). Interestingly, one of the two lateral contours, which is enclosed in the purple frame of Figure 3c,d, was not entirely bonded to the specimen’s core along its length. It is worth observing that the printer adopted a specific printing strategy: infill beads were deposited by alternating zero or near-zero gaps to wider gaps (as can be appreciated from the close-up image in Figure 3e). Therefore, voids in the specimen cross-sections were mainly located between the pairs of beads.

By transversally sectioning either the W090 and the N090 specimens with a plane intersecting both the 0° and 90° beads (type A-A section of Figure 3), the cross-section displayed a relatively low void content (see Figure 3d), whereas when the sectioning plane intersected exclusively the 0° beads (type B-B section of Figure 3), the resulting cross-section was highly discontinuous with a high porosity (see Figure 3c).

Sets of 10 binarized slice framing type A-A and B-B cross-sections of the N090 and W090 specimens were taken at discrete locations along the specimens’ gauge lengths, and they were then analyzed by ImageJ (version 1.53) software package [31], where the white pixels were counted as a resistant area. From these measurements, it emerged that the average cross sectional void fractions for the type A-A section were about 32% and 24%, while those of the type B-B section were about 5.3% and 2.8% for the N090 and W090 specimens, respectively. It is worth nothing that, from these measurements, the minimum recorded effective resistant area even dropped down to 65.2% and 71% of the nominal specimen cross-sectional area (which was defined as the width multiplied by thickness) for the N090 and W090 specimens, respectively.

The layer morphology and the printing strategy of the ±45° specimens were very similar to those of the 0°/90° ones (see Figure 4b). A similar highly discontinuous morphology was observed for the sectioning planes at ±45°, as shown in Figure 4d. The measurement of the cross-sectional void fraction for the type C-C section of the N45 and W45 specimens was carried out in a similar fashion as described for the 0°/90° specimens. The average cross-sectional void fractions amounted to 7.5% and 9% for the N45 and W45 specimens, respectively.

**Figure 5:**
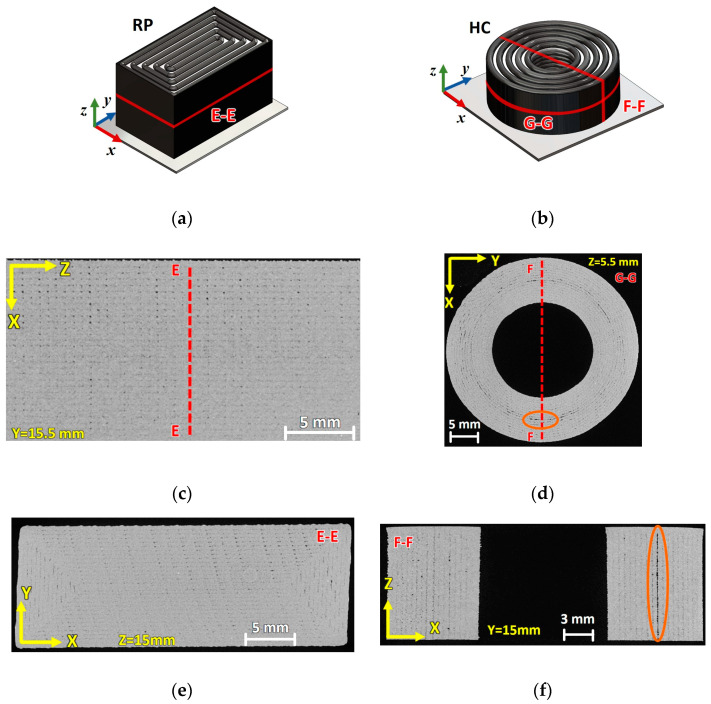
The geometry of the (**a**) RP and (**b**) HC specimens highlighting the reference frame, slicing planes (E-E, F-F and G-G), and µCT slices of the (**c**) X-Z and (**e**) X-Y layers (E-E cross section) of an RP specimen, as well as the (**d**) X-Y (G-G cross section) and (**f**) X-Z layers (F-F section) of an HC specimen (the voids of which are framed in orange boxes).

Slices extracted from the μ-CT scan of a pristine RP and HC specimen are shown in Figure 5. Figure 5a,b highlight the planes over which the μ-CT slices of the specimens were extracted. The morphology of the RP specimen meso-structure was relatively homogeneous, although small inter-beads voids were still present (see Figure 5c,e). A larger extension of the inter-bead voids was observed in the HC specimen (see Figure 5d,f). Voids were particularly concentrated between the outermost and innermost beads of the inner and outer contours, respectively, as shown in Figure 5d,f. Nevertheless, the overall morphology of the inner structure of the HC specimens was compact.

Cross-sectional void fraction measurements of the RP and HC specimens were performed by considering 10 slices of the type E-E and F-F sections along the specimen’s height. The average cross-sectional void fractions amounted to 2.1% and 2.8% for the RP and HC specimens, respectively.

### 3.2. Water Absorption

**Figure 6:**
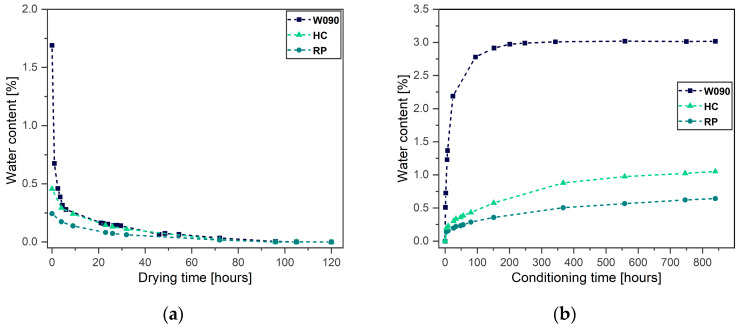
Percentage of the specimens’ water content evolution over (**a**) drying time and (**b**) conditioning time.

Figure 6a shows the evolution of the water content of the monitored specimens of each geometry over the drying time. The water content c was computed as follows:

(3) $$c=\frac{m\left(t\right)-m_{dry}}{m_{dry}}\times 100,$$

where $m\left(t\right)$ is the mass evolution over time, which was measured at prescribed time intervals, and $m_{dry}$ is the dry mass of the specimens.

The W090 specimen initially retained the greatest water content at about 1.7%. Its water desorption kinetics was the highest among the considered specimens. The initial water content of the compression specimens was heterogeneous. However, no control over the environmental exposure of such specimens before drying was exerted. After about 72 h (3 days) the water content in all the specimens did not vary appreciably anymore. Therefore, the drying procedure could be reasonably limited to that time span.

Figure 6b shows the evolution of the water content of the specimens of each geometry over the absorption time. The W090 specimen displayed the highest water absorption kinetics, as expected. After approximatively 250 hours, it reached the saturation at equilibrium with the specific environment of almost 3%.The absorption rate of the HC specimen was higher than that of the RP one. Within the same time window, the water contents of the HC and RP specimens amounted to 1.05% and 0.64%, respectively. Normally, a conventionally manufactured 3 mm-thick polyamide 6 specimen reaches the saturated water content in equilibrium with the same environment (room temperature and 50% relative humidity) in months [32].

### 3.3. Tensile Tests

**Figure 7:**
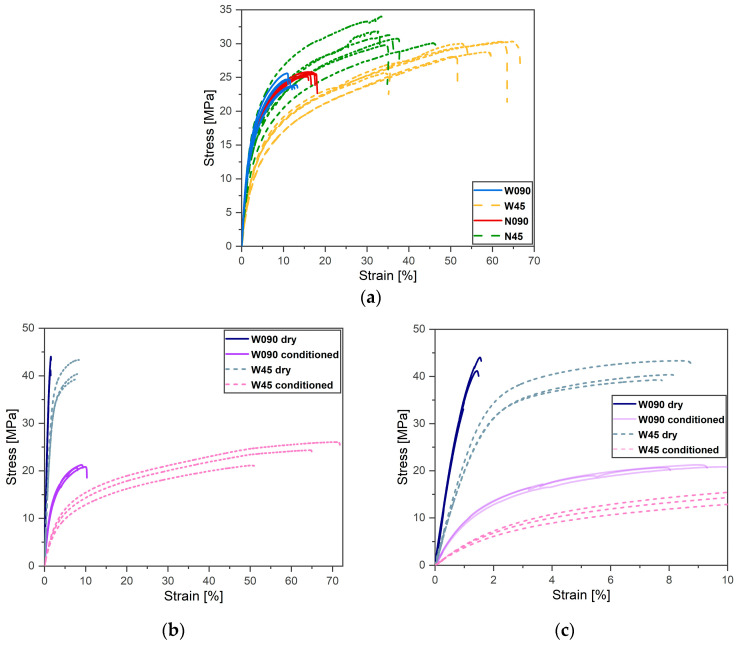
The tensile stress–strain curves of (**a**) the specimens belonging to the first printed batch, (**b**) the specimens belonging to the second printed batch (a part of the present curves is shown in [22]), and (**c**) a part of (**b**) in the initial strain range.

Figure 7 showcases the stress–strain curves resulting from the tensile tests. Figure 7a shows the results related to the first batch of specimens, which were tested at their current water content. The four specimens’ configurations (N090, N45, W090, and W45) retained distinct constitutive relations, thus meaning that both the specimen width and the raster orientation significantly affected the specimen properties. In general, the ±45° specimens showed a stronger and more ductile behavior than the 0°/90° ones. 

Considering the ±45° raster orientation, the larger width resulted in a 33% reduction in the Young’s modulus, which ranged from the 812 MPa of the N45 specimens to the 542 MPa of the W45 ones. For the 0°/90° raster orientation, the Young’s modulus seems insensitive to the specimen’s width instead. The N090 and the W090 specimens indeed displayed a comparable modulus of 981 MPa and 967 MPa, respectively. However, the UTS of the N090 specimens was 25.6 MPa, which was slightly higher than the 24.3 MPa of the W090 ones. Similar considerations held true for the ±45° raster orientation: the UTS of the N45 specimens was 31.3 MPa and the W45 ones was 29.2 MPa. A consistent 20% reduction in the UTSs was observed by comparing the ±45° and the 0°/90° specimens of the same width. The strain at failure (maximum strain) was higher for the N090 than for the W090 specimens (16.7% versus 12.1%), but lower for the N45 than for the W45 specimens (36.8% versus 56.6%). In accordance with the literature [8,14], by comparing the ±45° and 0°/90° specimens of the same width, the strain at failure variations of about 450% and 120% were observed for the large and narrow specimens, respectively. It is, however, worthwhile to underline that the failure strain of the 0°/90° specimens coincided with that of the 0°-oriented material beads.

The results of the tensile tests performed on the specimens belonging to the second batch, which were tested dry and conditioned, are reported in Figure 7b,c. Figure 7c shows a zoomed-in image of the information shown in Figure 7b so as to provide an more detailed visualization of the behavior of the dry specimens. The Young moduli, the ultimate tensile strength (UTS), the strain at failure, and the water content of the specimens belonging to the first and second batches are reported for each specimen configuration in Table 2.

A more compliant behavior of the ±45° specimens compared to the 0°/90° ones was also observed in these tests. On the one hand, the dry 0°/90° specimens behaved linearly up to failure, thereby showing an almost macroscopic perfectly brittle behavior with a strain at failure of about 1.7%. On the other hand, the conditioned ±45° specimens displayed limited linear behavior and large deformations, i.e., they consistently failed at strains higher than 50%.

The Young’s moduli were highly sensitive to the material water content: reductions of 75% and 80% were observed due to a water content of 3%. The values ranged from dry moduli of 4 GPa and 2.2 GPa to conditioned ones of about 1 GPa and 0.4 GPa for the W090 and W45 specimens, respectively. For similar FFF-ed SCFR-PA6 ±45° rectangular specimens with four contour beads, Nikiema et al. [15] reported a 70% Young’s modulus reduction when investigating dry to conditioned material with a water content of 2.3%.

The properties of the W090 and W45 specimens belonging to the first batch were close to those of their conditioned counterparts of the second batch. Therefore, it was reasonable to assume that, due to the strong hydrophilic nature of PA6 and the large porosity of the W45 and W090 specimens, the material water content at testing was close to 3%.

The 0°/90° and ±45° specimens displayed Young’s modulus reduction due to the increased water content of about 75% and 80%, respectively. 

For both raster orientations, the dry specimens showed UTS values that were about twice as much as those of the conditioned ones. The UTS of the conditioned 0°/90° and ±45° specimens were 21 MPa and 24 MPa, while values of 43 MPa and 41 MPa were observed for the dry specimens, respectively.

### 3.4. Compression Tests

**Figure 8:**
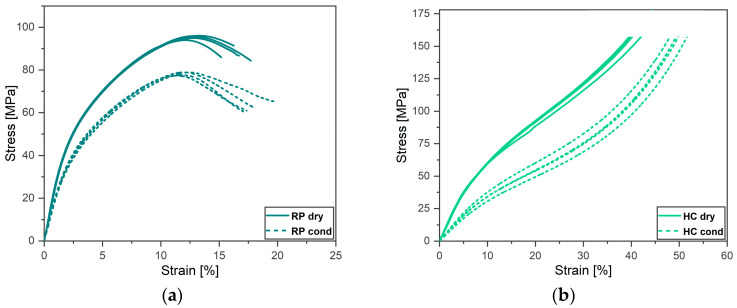
Compressive stress–strain curve of the dry and conditioned (**a**) RP specimens and (**b**) HC specimens.

In this work, the stress–strain curves resulting from the compression test are reported in Figure 8 in terms of their absolute values. 

The elastoplastic response of the RP specimens to the compressive load was characterized by noteworthy strain hardening, and this was regardless of the specimens’ water content, as shown in Figure 8a. However, a smooth transition from the linear elastic to the non-linear plastic region was observed.

The dry RP specimens displayed higher compressive strength (here taken as the maximum stress) than their conditioned counterpart; these were, respectively, equal to 95 MPa and 78 MPa. In both conditions, the maximum stress was reached at a comparable strain of about 12%. A 25% decrease in the longitudinal compressive modulus was observed due to the specimen’s water content of 1.05%, which ranged from 2.8 GPa to 2.16 GPa. 

The response of the dry and conditioned HC specimens to a compressive load, after an initial linear-elastic region, was overall a non-linear plastic, as shown in Figure 8b. Strain hardening occurred after yielding, and the slopes of the curves continuously varied as the strain increased. As in the case of the RP specimens, no clear yield point was observed for both testing conditions. Interestingly, about a 50% decrease in the transverse compressive modulus was observed due to the increased water content of 0.64%, which ranged from 0.8 GPa to about 0.4 GPa.

The water content, the compressive moduli, the compressive strengths, and the strain at which the maximum stress was reached, $\varepsilon_{str},$ are reported for the dry and conditioned RP and HC specimens in Table 3.

### 3.5. Failure Analysis

#### 3.5.1. Tensile Failure

**Figure 9:**
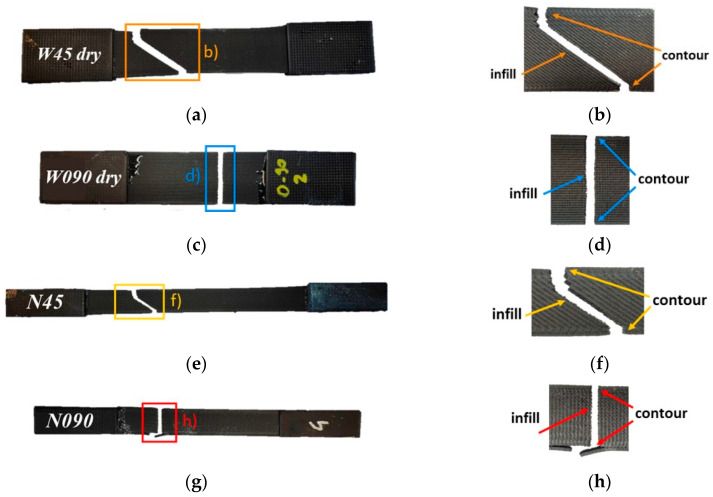
Picture of failed (**a**) W45 dry, (**c**) W090 dry, (**e**) N45, and (**g**) N090 representative specimens, as well as (**b**,**d**,**f**,**h**) close-up images of the respective fracture planes.

Figure 9 shows one failed sample for each specimen configuration. According to the literature [14,27], the failure plane of all the 0°/90° specimens should be perpendicular to the specimens’ longitudinal axis, i.e., perpendicular to the 0° beads (see Figure 9c,d,g,h). The fracture surface of all the ±45° specimens was oriented at either +45° or −45° in the infill region, where the raster orientation was ±45°. However, instead, it was noted as tending toward preserving an orientation perpendicular to the specimens’ longitudinal axis at the contour of the cross section, where the beads were oriented at 0° (see Figure 9a,b,e,f). For all the specimens, failure occurred in one of the sections with a reduced resistant area (see Section 3.1).

**Figure 10:**
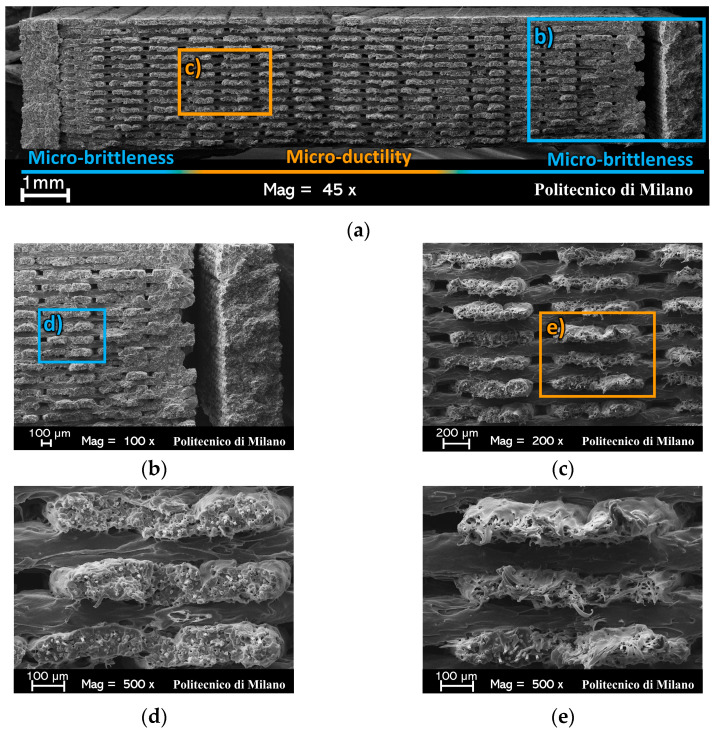
SEM observations of (**a**) the entire fracture surface, (**b**) micro-brittle, and (**c**) micro-ductile regions of a N090 specimen. Furthermore, (**d**) and (**e**) are close-up images of (**b**) and (**c**), respectively.

**Figure 11:**
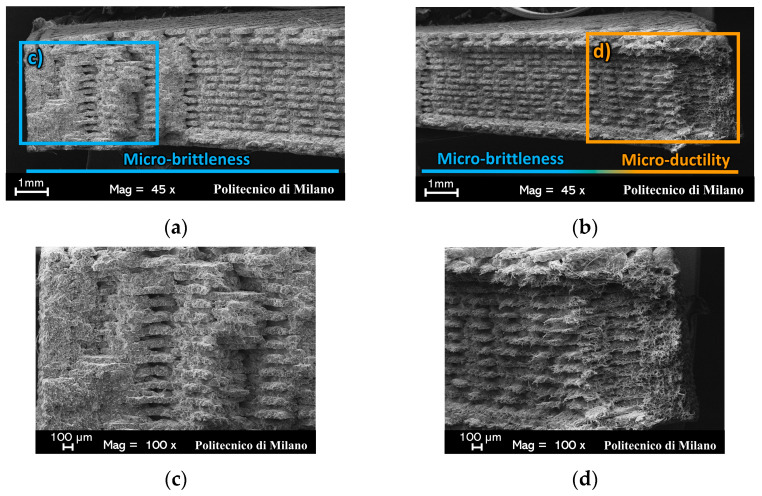
SEM observations of the fracture surface of a N45 specimen split into (**a**) a left side and (**b**) a right side because of perspective. Furthermore, (**c**) and (**d**) are close-up images of (**a**) and (**b**), respectively.

Figure 10 and Figure 11 show SEM images of the fracture surfaces of N090 and a N45 representative specimens (which were split in two halves due to the perspective distortions caused by the 45° inclination of the fracture surface). Their morphologies are presented in Figure 3 and Figure 4, respectively. 

Figure 10a shows an intra-bead at a 0° and at a 90° failure. This suggests that the effective resistant section of the 0°/90° specimens was provided by the 0° beads only, as quantified in Section 3.1. Similar considerations held for the ±45° specimens: Figure 11a,b evidence an intra-bead at a +45° failure and inter-beads at a −45° failure in the infill region.

For both raster orientations, two different micromechanical failure mechanisms could be inferred from the appearance of the broken beads. A limited number of beads experienced a micro-ductile failure, where the polyamide matrix underwent large plastic deformation before failure [33]. This fibrillation due to the crazing of the polyamide matrix resulted in highly elongated polyamide fibrils, which are visible in Figure 10c,e and Figure 11d. Conversely, the remaining beads of the cross-section showed minimal to no plastic deformation (see Figure 10b,d and Figure 11c); thus, they experienced a micro-brittle failure [33].

The micro-ductility and micro-brittleness of the specimen fracture surfaces was a result of the failure occurring at different strain rates [33]. At a low-strain rate, large deformations are associated with the confined micro-ductile region. Standing to reason, damage accumulates locally there, thus leading to the onset of failure. Once this region fails, the remaining specimen cross-section could not sustain the applied load any longer; thus, it suddenly failed. The high strain-rate failure of the specimen resulted in an extended micro-brittleness of the cross section.

It was thus possible to locate the failure onset region for each specimen, as analyzed by SEM. The 0°/90° specimens displayed a distributed network of 0°-oriented beads throughout their cross-section, where each of them could be a potential site for the onset of the specimen’s failure. Instead, the observed micro-ductility always included one of the two contours of the ±45° specimens (see Figure 11b). In accordance with their preferential alignment with the load, the 0°-oriented contour beads were stiffer than the infill ±45°-oriented ones. Thus, significantly higher stresses developed in the contour beads than in the infill ones. It was thus likely that the localized damage accumulated in the beads of the contours. This caused them to fail earlier than the rest of the specimen’s cross-section, most probably at the same strain level that caused the failure of the 0° beads in the 0°/90° specimens.

Upon the contour beads’ failure, the stress redistributed among the network of the ±45°-oriented beads in the infill. At this stage, the damage locally accumulated in the infill beads adjacent to one of the two contours, as supported by the pronounced micro-ductility of those beads in Figure 11b,d. Only once the beads in this confined infill region failed, did the sudden failure of the specimen occur in a brittle fashion. The beads of one out of the two contours of the ±45° specimens, shown in Figure 11a, displayed micro-brittleness since the high strain-rate fracture ran over a plane that was perpendicular to the ±45° beads in the infill and perpendicular to the 0° beads in the contours.

Overall, failure proved to be a matrix-dominated phenomenon; one that was localized in the contour beads of the ±45° specimen and in the confined ductile regions of the 0°/90° ones. No broken fibers were observed, while some were pulled out.

#### 3.5.2. Compressive Failure

**Figure 12:**
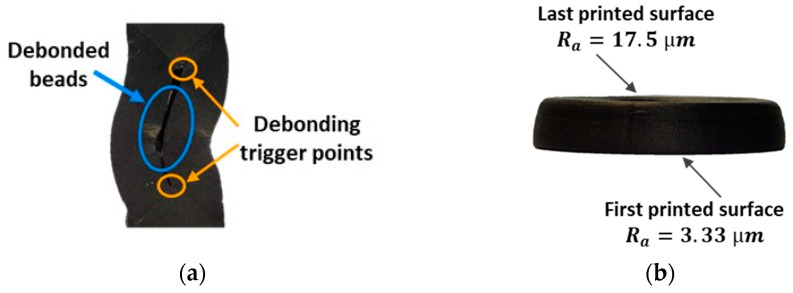
Failure morphologies of an: (**a**) RP and (**b**) HC specimen.

Figure 12a shows a representative RP specimen upon compression. Extensive debonding characterized its failure morphology. Inter-beads debonding interested the midsection of the specimen’s width in its gauge length (see Figure 12a). There, the left and right beads of the innermost contour loop met. This was possibly due to the poor adhesion that occurred between those mating beads while printing. Most likely, the onset of debonding was favored by the specific bead deposition strategy that was adopted by the printer. The sharp corners of the wedge-like regions (see Section 2.1.2) could act as preferential sites for stress concentration, hence promoting the local cleavage between adjacent beads. The propagation of the inter-bead debonding split the specimens’ gauge length into two halves. Each half started to behave as an individual load-bearing element with a reduced resistant area. The slenderness ratio of the individual column increased; therefore, the chances of buckling instability taking place also increase. A similar failure mechanism is known to occur in structural composites where a compressed surface layer detaches from its adjacent layer and buckles under a sufficiently large compressive load [34].

Figure 12b shows a representative HC-compressed specimen. Interestingly, the deformed shape of all the HC specimens upon compression was slightly similar to a truncated hollow cone. The first deposited surface, i.e., the one that lied on the building platform, underwent a larger radial deformation than the last deposited one, i.e., the free flat surfaces at the end of the print. This suggested that compression occurred under two different friction conditions at the interfaces between the top and bottom surfaces of the specimens and the compressive plates. The frictional forces that developed at the specimen–plate interface surfaces prevented the free lateral deformation of the material at those specific locations. The magnitude of these forces was found to be proportional to the roughness of the mating surfaces.

Therefore, surface roughness measurements were performed on the first and last deposited surfaces of two HC specimens. The measurements were performed according to the ISO 4288:1998 standard [35] using a Mahr PGK contact profilometer. The radius of the stylus was 2 µm. For every surface type, the measurements were performed over three radial paths along the specimens’ annulus thicknesses, which were spaced at a constant angle of 120°. The average surface roughness *R_a_* was taken as the average value of the three measurements along the prescribed paths. The average *R_a_* values were 3.33 µm and 17.5 µm for the first and last deposited surfaces, respectively. As a result, larger frictional forces developed at the interface between the rougher surface, i.e., the last deposited one, and the compression plate, thus locally providing a stronger opposition to the material radial deformation.

## 4. Discussion

### 4.1. Water Absorption of the Onyx Specimens

The large extent of the meso-structural voids made the W090 tensile specimens porous, thus increasing the material surface exposed to the environment; even the inner beads virtually experienced the external environment. Porosity might speed up the water absorption rate. It is likely that the absorption process for that specimen did not occur across the specimen’s thickness, but rather through each individual material bead. Consequently, the water absorption rate of the FFF-ed W090 specimen, which was printed using the previously specified set of parameters, was higher than that of their conventionally manufactured homogeneous counterparts. Celestine et al. [36] and Lay et al. [37] agreed on the appreciably higher-water absorption rate of the polyamide 6 FFF-ed specimens compared to that of the injection-molded ones. Despite the lower absorption rate of the SCFR-PA6 material compared with that of the neat polyamide 6, both attained a similar water content at saturation [38].

The water absorption kinetics of the HC and RP compression specimens were by far lower than that of the W090 specimen. The difference in the absorption and desorption rate among the W090, HC, and RP specimens was justified by the different specimens’ morphology. The low void content retained by the HC and RP compressive specimens makes them similar to homogeneous “bulk” materials. Moreover, the higher surface-to-volume ratio of the HC specimens, which was equal to 0.76, compared to that of the RP ones, which was about 0.30, resulted in a faster water absorption process in the considered timespan. However, the surface-to-volume ratio alone does not justify the faster water absorption of the W090 specimen. This parameter for the W090 specimen, indeed, was 0.75; thus, it was close to that of the HC specimen. Therefore, the large extent of porosity, which was retained by the W090 specimen, played a pivotal role in the water absorption process. 

### 4.2. Tensile Behavior of the Onyx Specimens

The wide and narrow specimens were printed with a constant contour area; therefore, their width directly affected the infill-to-contour area ratio of the specimens’ cross-sections. Despite this, the core and contour beads, both oriented at 0° (in parallel to the load), equally contributed to the overall stiffness of the 0°/90° specimens. The tensile modulus of the N090 specimens, indeed, was not significantly different from that of the W090 ones. However, the tensile modulus evaluated from the 0°/90° was hardly representative of that of the material bead. The 90° beads likely cooperated with the 0° ones to determine the stiffness of these specimens. Conversely, there were no 0°-oriented beads present in the infill region of the ±45° specimens. This firstly justifies the higher modulus of the W090 and N090 compared to that of the N45 and W45 specimens. Secondly, it confirmed the significant contribution of the 0°-oriented contour beads to the stiffness of the ±45° specimens. The lower the core-to-contour area ratio was, the more significant this contribution was. The tensile modulus of the N45 specimens was indeed considerably higher than that of the W45 ones. 

Water molecules act as softener agents for polyamides. The bond between the softened polyamide matrix and the short carbon fibers weakens [15]. As a result, the stress transfer from the matrix to the reinforcing fiber phase loses its effectiveness. Macroscopically, this translates into the more compliant and weaker behavior of the conditioned W090 and W45 specimens compared to that of their dry counterparts.

All the tensile specimens failed in one of the cross-sections with a reduced resistant area (see Section 3.1). This posed doubts on the effective stress that leads the specimens to failure. Considering the 0°/90° specimens as an exemplificative case, their strengths, collected in Table 2, were evaluated as the failure load divided by the specimen’s nominal resistant area. However, their effective resistant areas in the failure sections could be even 65% of the nominal ones (see Section 3.1). Therefore, their actual strength should be computed based on their effective resistant area. This suggests that the strength obtained from analysis in terms of nominal specimen dimensions of the experimental data was not representative of the actual stress that led the material beads to failure. 

Similar considerations might extend to the case of the ±45° specimens, although the presence of the contours and the raster orientation affect the stress distribution in the infill regions and in the contour beads. Moreover, longitudinal and transverse force components originate because of the ±45° misalignment between the raster and the loading direction. A complex stress state consisting of shear and normal stresses likely develops in the infill beads, which makes the analysis of the ±45° specimens in terms of actual dimensions of the cross-section even less immediate.

**Figure 13:**
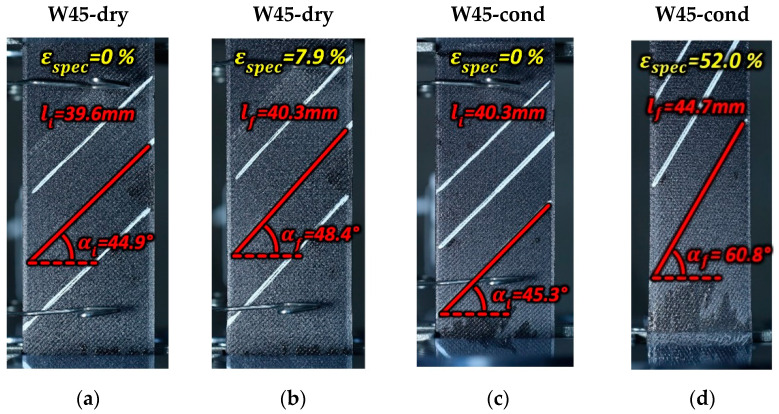
Image analysis of the core bead length of the dry-conditioned W45 specimens that were (**a**,**c**) unloaded and (**b**,**d**) those just before failure. (Pictures partially taken from [22]).

It is remarkable to mention that, as the tensile loading proceeded, a progressive re-alignment of the ±45°-oriented beads toward the loading direction was observed. It can be suggested that, for the ±45° specimens, the failure of the infill occurred when the longitudinal strain of the ±45° beads equaled the strain value at which the 0° beads of the 0°/90°specimens failed. An assessment of this hypothesis was made by evaluating the length of reference surface of the 45° beads in the dry and conditioned W45 specimens through image analysis. First, when they were unloaded (see Figure 13a,c) and then just before their failure (see Figure 13b,d).

While the specimen’s strain $\varepsilon_{f,spec}$ was measured from the tests, the strain at failure of the reference inclined beads ${\bar{\varepsilon }}_{f,bead}$ was computed as the difference between the bead’s final and the initial length ($l_{f}$ and $l_{i}$) over its initial length. The strains at failure of the reference beads were 1.75% and 10.7% for the dry and conditioned W45 specimens, respectively. Hence, they were present within the scatter of the failure strains of the 0° beads of the W090 dry and conditioned specimens (reported in Table 4). Therefore, despite the huge difference in strain at failure between the 0°/90° and ±45°specimens, which is mentioned in Section 4.1, the infill beads (oriented at ±45°) of the ±45°specimens and the 0° beads of the 0°/90° specimens failed at comparable strain levels. The parameters involved in the computation of ${\bar{\varepsilon }}_{f,bead}$ are also reported in Table 4.

As a result, the ±45° meso-structure afforded elevated specimen’s longitudinal elongations despite the limited strain experienced by each ±45°-oriented individual material bead. The higher the material water content, the larger the admissible elongations of the specimens and the degree of raster re-alignment toward the loading direction are. It is worth remarking that this phenomenon also hugely affected the fatigue response of the specific combination of material, the meso-structure, and the specimen geometry considered, as reported by the authors in [22].

It was suggested at the end that the tested 0°/90° and ±45° specimens delivered the specific tensile properties of the distinctive combination of the meso-structures, geometries, and dimensions considered. Characterizing the longitudinal tensile properties of the underlying material would be preferrable for testing the narrow prismatic specimens made of concentric contour beads only. In this way, a unidirectional 0° deposition of the beads in their gauge length can be obtained. However, these specimens should be sufficiently long to confine the critical wedge-like regions in a limited portion of the specimens’ gripping area, thus alleviating any possible effect of a printing path-induced stress concentration. Tensile tests should be supported by a µ-CT investigation of the specimen’s meso-structure morphology, and the experimental data should be analyzed in terms of the effective resistant area.

### 4.3. Compressive Behavior of the Onyx Specimens

The results of the compression tests evidenced the expected anisotropic properties of the FFF-ed Onyx material. The longitudinal and transverse compressive moduli, respectively evaluated from the RP and HC specimens, could be reasonably considered reliable estimations of the actual material elastic properties. 

Due to the different roughness of the first and last printed surfaces of the HC specimens, dissimilar frictional forces developed at the interfaces between these specimen surfaces and the compression plates. This resulted in a local uneven radial deformation of the material. The barreling phenomenon (see Figure 12b) also originates from the effect of those interface frictional forces. In that case, the material close to the top and bottom specimen surfaces radially deforms less than that far from them, due to the action of similar interface frictional forces.

Given the similarity and the common cause of the two phenomena, the effect of barreling, as well as the effect of the slight asymmetric radial deformation on the accurate evaluation of the SCFR-PA6 transverse compressive modulus, should be minimized by the small aspect ratio of the HC specimen. Sandblasting the specimen’s surfaces to make their roughness uniform would lead to a more even radial deformation of the material.

Inter-bead debonding splits the gauge length of the RP specimens into two halves. Most likely, these two semi-columns of a reduced resistant area buckle at the failure of the specimen. Therefore, the SCFR-PA6 longitudinal strength might not be properly evaluated by testing the RP specimen. However, the evaluation of the longitudinal compressive modulus, which is carried out in the early stages of the compression test where debonding does not take place, should be reliable.

In any case, for an accurate evaluation of the material longitudinal strength, it is suggested to test a specimen whose geometry combined with the bead’s deposition path ensures full alignment of the beads in the loading direction. Any possible critical region that could trigger the debonding of adjacent beads should be avoided. Even if cylindrical specimens are considered, a similar conical wedge like structure as the one obtained in the RP specimens would be printed. This challenges the end user to characterize the material properties using ready-to-test FFF-ed specimens. Alternatively, the possible critical regions of the specimens should be removed. This, however, does not take full advantage of the FFF technology for one-step manufacturing of adequate specimens for material properties characterization.

## 5. Conclusions

The static mechanical behavior of the FFF-ed SCFR-PA6 material was investigated with respect to the meso-structure morphology and water content of the specimens tested. The test specimens were custom-designed to comply with the technological limitations of a commercially available FFF printer. The μ-CT scans revealed that a large degree of inter-bead voids concentrate in the infill region of the specimens, even when a 100% solid infill is selected. Even when contouring instead, it always resulted in a compact and homogeneous-like structure. Overall, this affected the effective resistant area of the specimens and their water absorption kinetics. The specimen’s porosity favoured the material water absorption rate: SCFR-PA6 attained its saturated water content in a humid environment (23°C and 50% relative humidity) of 3% in about 10 days.

According to the findings of the present work, assessing the contribution of each individual material bead to the overall mechanical behavior of a specific bead’s layout, i.e., 0–90° or ±45° raster orientation, is demanding. This suggests that an analysis of the test outcomes compels the implementation of a multi-scale model to relate the mechanical properties of the individual material bead to those of the specimen.

Consequently, one might think that the mechanical properties, which were evaluated from 0°/90° and ±45° specimens, refer to the specific meso-structure investigated, i.e., that which is indicative of the mechanical behavior of that peculiar internal bead’s arrangements. Hence, this information would be useful for designing a part that displays a bead’s layout, which resembles that of the tested specimen. However, while the tensile properties of the 0°/90° specimens were almost insensitive to the width of the infill region, those of the ±45° specimens dramatically depend on it. Therefore, the information extracted from the test specimens referred to the specific combination of their meso-structure, geometry, and dimensions.

Long and narrow prismatic specimens, i.e., those that were FFF-ed with concentric contour beads only, were instead suggested for the characterization of the longitudinal tensile properties of the material beads. However, the experimental data should be analyzed in terms of the actual specimens’ resistant cross-sections. 

The HC specimen with a low aspect ratio looked suitable for evaluating the material’s compressive transverse modulus. The RP specimens allowed the material’s longitudinal compressive modulus to be reasonably evaluated. However, for an accurate longitudinal compressive strength evaluation, unidirectional specimens should be printed by avoiding any critical area that promotes the onset of beads debonding. Otherwise, such critical areas should be removed by an additional manufacturing step. 

## Figures and Tables

**Table 1:** Parameters used for the µ-CT scanning of tensile and compressive specimens.

Specimen	Voltage [kV]	Current [A]	Voxel Side [µm]	Focal Spot [µm]	Magnifying Factor	Consecutive Volumes
W090–W45	90	70	15.4	15.3	6.5	2
N090–N45	77	100	7.8	7.7	12.7	4
RP-HC	120	110	24.5	13.2	4.1	1

**Table 2:** Results of the tensile tests, where “±” indicates a standard deviation (a part of the present dataset was shown in [22]).

Tensile Specimens	Water Content [%]	Young’s Modulus [MPa]	UTS [MPa]	Failure Strain [%]
1st batch				
W45	Not measured	542 ± 24	29.2 ± 1.7	56.6 ± 10
N45	Not measured	812 ± 77	31.3 ± 1.4	36.8 ± 4.4
W090	Not measured	967 ± 27	24.3 ± 1.3	12.1 ± 0.7
N090	Not measured	981 ± 36	25.6 ± 0.5	16.7± 0.5
2nd batch				
W45-dry	-	2152 ± 82	41.0 ± 1.7	8.2 ± 0.4
W45-cond	2.95	412 ± 11	23.9 ± 2.5	62.7 ± 12.8
W090-dry	-	4010 ± 69	43.1 ± 1.3	1.7 ± 0.25
W090-cond	3.02	1074 ± 45	20.9 ± 0.3	10.5 ± 0.8

**Table 3:** Results of the compressive tests, where “±” indicates a standard deviation.

Specimen	Water Content [%]	Compressive Modulus [MPa]	Compressive Strength [MPa]	$\varepsilon_{str}$ [%]
RP-dry	-	2799 ± 99	95.1 ± 0.7	12.8 ± 0.5
RP-cond	1.05	2160 ± 71	78.2 ± 0.7	11.9 ± 0.4
HC-dry	-	797 ± 31	-	-
HC-cond	0.64	410 ± 32	-	-

**Table 4:** The quantities involved in the computation of the failure strain of the surface 45° beads. (Part of the present dataset was shown in [22]).

Specimen	$l_{i}$ [mm]	$l_{f}$ [mm]	$\alpha_{i}$ [°]	$\alpha_{f}$ [°]	${\bar{\varepsilon }}_{f,spec}$ [%]	${\bar{\varepsilon }}_{f,bead}$ [%]	Failure Strain [%]	
							W090-Dry	W090-Dry
W45-dry	39.6	40.3	44.9	48.4	7.9	1.75	1.7 ± 0.25	-
W45-cond	40.3	44.6	45.3	60.6	52.0	10.7	-	10.5 ± 0.8

## Data Availability

Data will be made available on request.
